# Overcoming barriers to mental health care: multimodal trauma-focused treatment approach for unaccompanied refugee minors

**DOI:** 10.1186/s13034-021-00404-3

**Published:** 2021-09-30

**Authors:** Carlijn M. van Es, Marieke Sleijpen, Merel E. Velu, Paul A. Boelen, Renate E. van Loon, Marjan Veldman, Nebil Kusmallah, Paula J. C. Ekster, Trudy Mooren

**Affiliations:** 1 Department of Clinical Psychology, Utrecht University, Utrecht, The Netherlands; 2 ARQ Centrum’45, partner in ARQ National Psychotrauma Centre, Diemen, The Netherlands; 3 Nidos, Utrecht, the Netherlands; 4 Ouder- en Kind Team Amsterdam, Amsterdam, the Netherlands; 5 Faculty of Social Sciences, Department of Sociology, Vrije Universiteit Amsterdam, Amsterdam, The Netherlands; 6 De Evenaar, Center for Transcultural Psychiatry, GGZ Drenthe Mental Health Care, Assen, The Netherlands

**Keywords:** Unaccompanied refugee minors, Trauma-focused treatment approach, Barriers, Treatment integrity, Feasibility

## Abstract

**Background:**

This study evaluated the feasibility of a short-term, multimodal trauma-focused treatment approach adapted specifically for unaccompanied refugee minors (URMs) in the Netherlands. This approach aims to overcome barriers to mental health care and to reduce symptoms of posttraumatic stress disorder (PTSD) and depression.

**Methods:**

An uncontrolled study was conducted, evaluating the main request for help, treatment integrity and feasibility, and the course of symptoms of PTSD (Children's Revised Impact of Event Scale-13) and depression (Patient Health Questionnaire modified for Adolescents).

**Results:**

In total, 41 minors were included in the study. Most participants were male (*n* = 27), predominately from Eritrea (75.6%) with a mean age of 16.5 (SD = 1.5). Minors mostly reported psychological problems, such as problems sleeping, and psychosocial problems, including worries about family reunification. Deviations from the approach were made to meet the current needs of the minors. Factors limiting the feasibility of the approach were often related to continuous stressors, such as news concerning asylum status.

**Conclusions:**

The results provide a first indication that this approach partly overcomes barriers to mental health care and emphasize the added value of collaborating with intercultural mediators and offering outreach care.

*Trial registration*: The study was registered in the Netherlands Trial Register (NL8585), 10 April 2020, Retrospectively registered, https://www.trialregister.nl/trial/8585.

## Background

Tailored psychosocial support for vulnerable groups, such as unaccompanied refugee minors (URMs), is of great importance. URMs are refugee children and adolescents under the age of 18, who have been separated from their primary caregivers [1]. In 2017, approximately 6000 URMs were under guardianship in the Netherlands, with the majority coming from Eritrea, Afghanistan, or Syria. In general, refugee minors are exposed to a variety of stressors before and during their flight, such as forced migration and other dangerous circumstances. After migration, these minors often continue to experience hardships, such as resettlement in a new and strange environment, racial discrimination, isolation, and insecurities concerning family reunification and their refugee status. The potentially traumatic events before and during the flight, as well as the continuous stressors related to the post-migration context can affect the mental health of these refugee minors [2–4]. URMs are considered one of the most vulnerable groups of refugees [5]. Studies indicate that, compared to accompanied minors, many URMs were exposed to more adverse events, such as sexual assault and violence [5, 6]. The vulnerability of URMs might be further enhanced by the separation from their caregivers and the lack of support that parental and family care can offer [7].

Altogether, URMs are at greater risk of developing mental health problems than accompanied minors. A substantial group of URMs develop symptoms of posttraumatic stress disorder (PTSD) and/or depression, which may become chronic [8]. As mental health problems during childhood and adolescence can be long-lasting, it is crucial to address these problems promptly [9]. Furthermore, the continuous stressors URMs face are likely to maintain their mental health complaints [8, 10]. For example, Eritrean URMs and their caregivers in the Netherlands stated that continuous stressors, such as worries about their family members, the complex family reunification procedure, and a lack of financial resources, highly affected their psychological functioning. URMs and their caregivers suggested that, as a result, URMs developed, or experienced an exacerbation of, sleeping problems, difficulties concentrating, and other health issues [11].

Although URMs have an elevated risk of developing mental health complaints, few studies have focused on interventions and programmes targeting these complaints. Most of these studies were qualitative or based on case descriptions [12, 13]. To our knowledge, only one randomized controlled trial (RCT) was conducted. This study showed that a trauma-focused group intervention for URMs, drawing on cognitive behavioural principles, improved self-reported symptoms of PTSD and depression, but not caregiver-reported symptoms [14]. Moreover, a case study and an uncontrolled pilot study on trauma-focused cognitive behavioural therapy (TF-CBT) yielded preliminary evidence that this therapy is feasible and may effectively reduce PTSD symptoms in traumatized URMs [2, 13]. Finally, a recent pilot study suggested it is feasible to implement Narrative Exposure Therapy (NET) for URMs﻿ [14, 15].

One possible reason for the lack of intervention studies is the number of barriers faced when offering trauma-focused interventions to URMs. One study indicated that although approximately 60% of the participating URMs reported a need for professional support, only 11.7% had actually received this help [16]. Barriers to the provision of adequate mental health care include individual barriers—such as linguistic differences, taboos concerning mental health care, lack of knowledge of the mental health system and mental health disorders, as well as a fear of stigma—and structural barriers—such as a lack of financial coverage for treatment and interpreters, and poor access to services [7, 12, 16–19]. We therefore face the challenge of diminishing these barriers and offering culturally sensitive and accessible interventions to this group of minors.

To overcome barriers to mental health care, we developed a short-term (approximately eight-session) multimodal trauma-focused treatment approach, specifically adapted for URMs. This approach, described in more detail below, includes CBT interventions to target continuous stressors and to alleviate symptoms of depression and (traumatic) stress. The treatment approach aims to overcome individual as well as structural barriers by (a) offering the treatment at the living location of the minors or another location of the minor’s choice, (b) collaborating with intercultural mediators (ICMs), who aided in understanding cultural and language differences between the minor and the therapist, and (c) conducting a flexible, multimodal treatment approach that allows the treatment sessions to focus on the minors’ current request for help.

This paper describes a pilot study designed to evaluate this trauma-focused treatment approach for URMs. Specifically, we explored (a) the main request for help among URMs in the Netherlands referred to the treatment approach; (b) the treatment integrity (the extent to which we were able to apply the approach as intended) and feasibility (whether the approach can be implemented successfully), and (c) the course of symptoms of PTSD and/or depression. The information collected during this pilot study was meant to help us in refining the treatment approach to meet the needs of URMs in the Netherlands adequately. The findings will also inform future efforts in studying treatment programmes for URMs.

## Methods

### Participants

Participants were URMs referred to the multimodal treatment approach by their legal guardian or general practitioner. Inclusion criteria were being (a) a URM living in the Netherlands under the guardianship of Nidos (the national guardianship institution for unaccompanied and separated children under the age of 18 in the Netherlands); (b) younger than 19 years old at referral; (c) from Eritrea, Syria or Afghanistan; and (d) with symptoms of PTSD and/or depression as reported by the legal guardian/general practitioner. Minors were excluded when pharmacological treatment or crisis intervention was required for acute suicidality or psychosis, as these issues were beyond the scope of the multimodal treatment approach. In these cases therapists or youth care professionals aided in finding adequate help.

As some URMs receive extended youth care after they turn 18, minors up to 19 years old could be referred for treatment. It was chosen to offer the treatment to minors from Eritrea, Syria or Afghanistan because the majority of URMs in the Netherlands came from these countries [20] and professionals working at Nidos perceived that minors from these countries experienced barriers to accessing regular mental health care. ICMs from Eritrea, Syria and Afghanistan were therefore trained to offer the treatment approach. Finally, as the treatment aims to address symptoms of PTSD and/or depression, only minors with reported symptoms were referred. No power analysis was conducted to estimate the number of participants as the aims of the current study were exploratory. Recruitment and inclusion ran from October 2017 until March 2020.

### Procedure

Guardians were informed about the intervention by youth care professionals working at Nidos. Guardians identified minors who experienced symptoms of PTSD and/or depression in addition to barriers to regular mental health care, such as low motivation for treatment, long waiting lists, and the idea that Western health care would not correspond with the URM’s needs and culture. The minors were consequently referred for the multimodal trauma-focused treatment approach. Next, a therapist and ICM visited the minor at their living location or another location of the minor’s choice, for an intake interview. The trauma-focused treatment approach then started. Assessments of psychological complaints and potentially traumatic events were carried out during the session following the intake interview (t_1_). Post-measurements of psychological complaints were conducted during the last session (t_3_). After each session, therapists filled in a list assessing elements of programme integrity (t_2_). After all treatments had been completed, all ICMs and therapists were asked to fill in a questionnaire to evaluate the trauma-focused treatment approach. The questionnaires were conducted online or over the telephone due to restrictions related to COVID-19.

To assure data integrity and compliance with ethical and juridical aspects, the study was reviewed by the Medical Ethics Committee of the Leiden University Medical Centre. The Committee stated the study did not require ethical approval as it intended to improve care as usual. Minors received verbal information on the study and provided verbal or written informed consent. For minors below 16 years of age, legal guardians also gave verbal or written consent. The assessments with the minors were part of the treatment evaluation and also used for clinical purposes. Participating therapists and ICMs gave online consent for the use of their data for research purposes.

### Measurements

All assessments of the minors were administered with help from an ICM. The Children’s Revised Impact of Event Scale-13 (CRIES-13) is available in several languages (www.childrenandwar.org). The Patient Health Questionnaire-9, modified for adolescents (PHQ-A) is available in English. When a minor spoke another language, the questionnaires were translated into this language by an ICM.

#### Demographic information

The following demographic information was collected: gender, age, country of origin, whether the minor came from a city or town, and whether the minor had any family members in the Netherlands.

#### Request for help

To map how URMs define their main problem and request for help, minors were asked to formulate their main problem.

#### Life events

During the second treatment session, therapists identified positive life events, adverse life events, and losses of loved ones the minors had been exposed to. They did so by laying down a lifeline, as is described in the Narrative Exposure Therapy for the treatment of traumatized children and adolescents’ protocol (KIDNET; described in more detail in the ‘Treatment and Therapist’ section). Minors were asked for their permission to share information concerning their treatment sessions, including the lifeline, with the researchers.

#### Programme integrity

To assess whether the treatment approach was performed as intended, two components of programme integrity, based on the conceptual framework of Carroll et al. [21] were assessed: exposure and adherence. Exposure was operationalized as the number of sessions per client and minutes per session. Adherence was operationalized as the extent to which therapists adhered to the specific components of the approach. The programme integrity list was completed by the therapist after each session. In addition, the programme integrity list included questions on the duration of the treatment approach, how therapists evaluated the collaboration with the ICM, the main theme of each session, whether a module protocol was used, whether the therapist deviated from the treatment approach, and why they deviated from the treatment approach.

#### Feasibility

After finishing all sessions, the therapists and ICMs involved in the study were asked to evaluate the multimodal trauma-focused treatment approach by filling in an online questionnaire. The questionnaire was based on a brainstorming session in which the researchers discussed the evaluation aims. It consisted of mostly open-ended questions focusing on positive aspects of the intervention and elements that could be improved or changed. Therapists and ICMs were also asked to arrange aspects of the approach according to their importance, based on their experience.

#### Symptoms of posttraumatic stress

All minors completed the CRIES-13, a 13-item tool measuring symptoms of posttraumatic stress in children aged 8 and older. The scale includes three subscales: intrusion, avoidance, and arousal. Items are rated on a 4-point scale rating 0 = *not at all,* 1 = *rarely,* 3 = *sometimes,* and 5 = *often*. A total score of ≥ 30 suggests an increased risk of PTSD [22]. The CRIES-13 is a valid measure of posttraumatic stress that has been used extensively among children exposed to war and with different cultural backgrounds [22, 23]. The Cronbach’s alpha in this study was good (0.81).

#### Symptoms of depression

To measure symptoms of depression, the PHQ-A [24] was used. The PHQ-A is a modified version of the PHQ-9. For example, ‘reading the newspaper’ in the PHQ-9 questionnaire was changed to ‘schoolwork and reading’ in the PHQ-A. The PHQ-A includes nine items rated from 0 (*not at all*) to 3 (*nearly every day*) with a time period of the past 7 days. In line with the PHQ-9, a total score of ≥ 10 was considered as the cut-off score for detecting depression [25]. Despite a few differences, the measure is mostly consistent with DSM-5 criteria for a major depressive disorder. The PHQ-9 is a validated, frequently used instrument measuring depression in adolescents [26]. The last question of the PHQ-A is on self-harm and suicidality. All therapists were licensed and trained to explore suicidality, and suicidality was also included in the training for ICMs. If a minor was suicidal, it was explained to the URM that their guardian and/or mentor would be informed. If needed, a child and youth psychiatrist could be consulted. Although the reliability and validity of the PHQ-A for refugee minors from different backgrounds have, to our knowledge, not yet been established, the PHQ-A has been shown to have acceptable psychometric properties when completed by Arabic-speaking adolescent refugees [27]. The Cronbach’s alpha in this study was acceptable (0.79).

#### Treatment and therapists

Before starting the present study, we performed a non-systemic evaluation of the multimodal trauma-focused treatment approach [28]. After offering the treatment to 33 Eritrean URMs, the therapists and researchers reviewed the approach. They noted that it focused mainly on traumatic events in the past, however, they emphasized the importance of tailoring the treatment to meet the minor’s individual needs, for example by focusing more on current, ongoing stressors. The approach was therefore adapted to allow for more flexibility. Moreover, the importance of involving the direct context of the URM (e.g., guardian and mentor) was emphasized by the involved professionals.

**Fig. 1:**
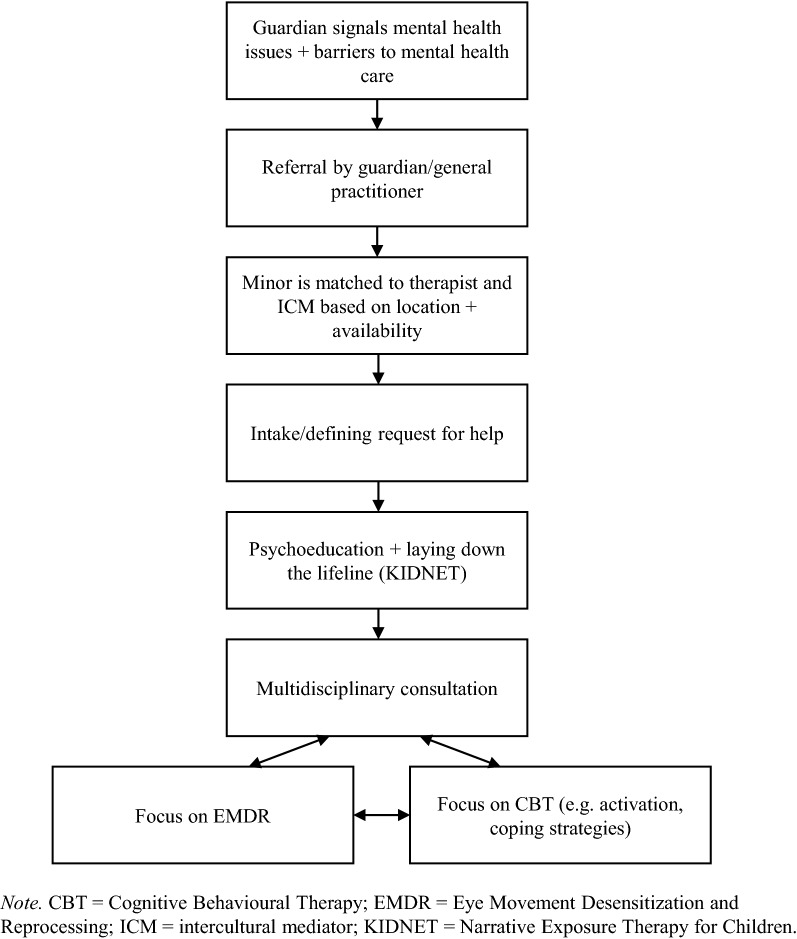
Treatment approach procedure

The procedure is presented in Fig. 1. URMs were offered the multimodal, culturally sensitive, trauma-focused treatment approach. The approach started with a clinical intake interview. During the intake, confidentiality and mutual expectations were discussed. During the following session, psychoeducation about PTSD symptoms and trauma-focused treatment were offered. As URMs are often unfamiliar with such treatments, and differences in explanatory models between the therapist and the minors might exist, considerable time was spent on psychoeducation. For example, therapists normalized symptoms by explaining how complaints might be linked to traumatic experiences in the past and continuous stressors. Additionally, the treatment rationale and potential side effects of the treatment approach were explained. Finally, the minors’ lifeline (as derived from KIDNET) was laid out, either by drawing or by laying down a rope and placing flowers for their positive experiences and stones for their negative experiences. Next, it was decided which intervention module suited the minor best. This was done during multidisciplinary consultation based on the request for help and complaints as reported by the minor. Each session lasted approximately 90 min. The final session focused on leave-taking. If more help was needed, the therapist discussed with the minor and the guardian what was needed and, if necessary and suitable, the therapist provided this. If therapists were not able to offer this support, they assisted the URM and their guardian to find suitable help. For example, therapists helped finding suitable mental health care nearby.

The treatment approach included different modules, including CBT interventions and elements of KIDNET (the lifeline) and Eye Movement Desensitization and Reprocessing (EMDR), to address continuous stressors, and symptoms of depression and (traumatic) stress. Culturally sensitive adaptations of the modules included collaborating with ICMs before and during the sessions. Before the sessions, how to offer psychoeducation in a culturally sensitive way was discussed, for example by using suitable metaphors. The core components of the treatment modules were not adapted.

##### NET

The lifeline was construed based on KIDNET [29] and served as a way to identify key adverse life events, positive life events, and losses of loved ones. Moreover, the lifeline provided insight into the link between complaints and traumatic events (a case conceptualization). NET is conditionally recommended by the American Psychological Association (APA) for the treatment of PTSD [30].

##### EMDR

URMs who had developed traumatic stress reactions as a result of a clearly defined traumatic incident received EMDR to address these issues. EMDR is a frequently used psychotherapeutic treatment [31] focused on weakening negative and strengthening positive cognitions associated with the traumatic event. A key element of EMDR is a dual attention task. Whilst the client focuses on an image of the traumatic memory, bilateral, rhythmic stimulation, for example evoking saccadic eye movements, is offered [32]. Oras et al. [33] suggested that EMDR is an effective treatment for traumatized refugee children and the guidelines of the National Institute for Health and Care Excellence (NICE) cited EMDR as a promising treatment for children and adolescents [34, 35]. After discussions with ICMs, therapists, professionals with experience working in Eritrea, and EMDR experts, it was decided to offer EMDR, because during EMDR minors do not have to verbalize all of their traumatic experiences, which is more often the case in other trauma-focused treatments, such as KIDNET. URMs might be unfamiliar with talking about their experiences and therefore might be hesitant to do so.

##### CBT

NICE recommends CBT for the treatment for depression [36]. During the intervention, CBT was offered, for example, by supporting URMs in increasing activities and improving social connections [37].

#### Therapists

The trauma-focused treatment approach was performed by therapists working at various mental health care institutions throughout the Netherlands. Therapists were recruited based on the network of the project team involved in designing the treatment approach. Therapists were all licensed mental health care workers and trained EMDR-therapists with multiple years of experience with minors from different cultural backgrounds. Therapists took part in multidisciplinary consultation as part of their regular work, as well as multidisciplinary consultation and supervision as part of the multimodal treatment approach. The motivation for therapists to take part in offering the treatment approach included the collaboration with ICMs, being part of a network of experienced psychologists, and working together to increase the understanding of what works for URMs in the Netherlands.

#### ICMs

ICMs were persons close to the URMs in cultural background and experience, who aimed to facilitate communication between the therapist and the minor. They interpreted language and offered information on the cultural background of the URMs. All ICMs followed a training focused on trauma, stress, and selfcare before providing the approach and were given the opportunity to participate in intermediate supervision sessions.

### Analysis

#### Main request for help

After an initial assessment of the main requests for help of the minors their requests were categorized into psychological problems, psychosocial problems, and somatic problems. All requests for help were related to these categories. In addition, a previous study indicated that URMs in the Netherlands often report psychosocial stressors and psychological complaints [11]. Moreover, refugees often present somatic symptoms [38].

#### Treatment integrity and feasibility

To evaluate programme integrity, exposure, and adherence were computed. Missing items were allowed on the programme integrity list. When percentages are reported, they apply to all questions that have been completed. Adverse events were categorized following the subsequent categories, in line with the World Health Organization World Mental Health Surveys [39]: [1] natural and man-made disasters and accidents; [20] combat, war, and refugee experiences; [6] sexual and interpersonal violence; [7] witnessing or perpetrating violence; and [8] death of a loved one. Being captured or held against one’s will was added as a category as more than 25% of minors reported experiencing this adverse event.

Data from the online questionnaires were analysed using the general inductive approach for analysing qualitative evaluation data [40] in the following steps. All steps were conducted in parallel (CvE and MeV). First, the text was read thoroughly. Second, specific text fragments related to the research questions were identified and labelled to create categories. Third, overlap and redundancy were reduced. Finally, the most important categories were identified. The categories were discussed among the two raters until a consensus was reached. During this process, an ongoing discussion was maintained with the rest of the research team.

#### Course of symptoms

Data of the pre- and post-treatment assessments of the CRIES-13 and PHQ-A were analysed using SPSS (version 27, IBM Statistics). For the CRIES-13, in line with Verlinden et al. [41], data were counted as missing if more than one item on a subscale was missing. For the PHQ-A, in line with prior studies on the PHQ-9 [42], data were counted as missing if more than two items were missing. Missing values on the PHQ-A were replaced by the mean of the completed items of the PHQ-A, as suggested by Kocalevent et al. [43] and missing items on the CRIES-13 were replaced by the mean of the completed items of the same subscale. A Chi-square test and independent t-test were conducted to evaluate whether any differences related to demographic information and baseline scores on the CRIES-13 and PHQ-A existed between participants who completed the questionnaires and participants who did not. To assess whether any changes occurred in symptoms of depression and PTSD, paired t-tests and Wilcoxon-signed rank tests were conducted. To evaluate clinical significance of change, we calculated the Reliable Change Index (RCI) for changes in symptoms of depression and PTSD, using the formula $\frac{t3-t1}{Sdiff}$ [44], with t_3_ referring to post-treatment assessments of the PHQ-A and CRIES-13, t_1_ referring to pre-treatment assessments of the PHQ-A and CRIES-13, and S_diff_ being calculated using the test–retest reliability coefficient of the questionnaires and the standard deviation of the pre-treatment scores. The test–retest coefficient of the CRIES-13 is 0.85 [45]. Kroenke et al. [46] found that the test–retest reliability of the PHQ-9 was 0.84. A calculated RCI larger than |1.96| indicated a clinically reliable change, with 95% certainty. The RCI resulted in numbers of minors improved, unchanged, and worsened from t_1_ to t_3_. In addition to the RCI, clinically significant change was further evaluating by assessing whether clients had obtained a score of < 30 on the CRIES-13, and a score of < 10 on the PHQ-A after the treatment approach.

## Results

**Fig. 2:**
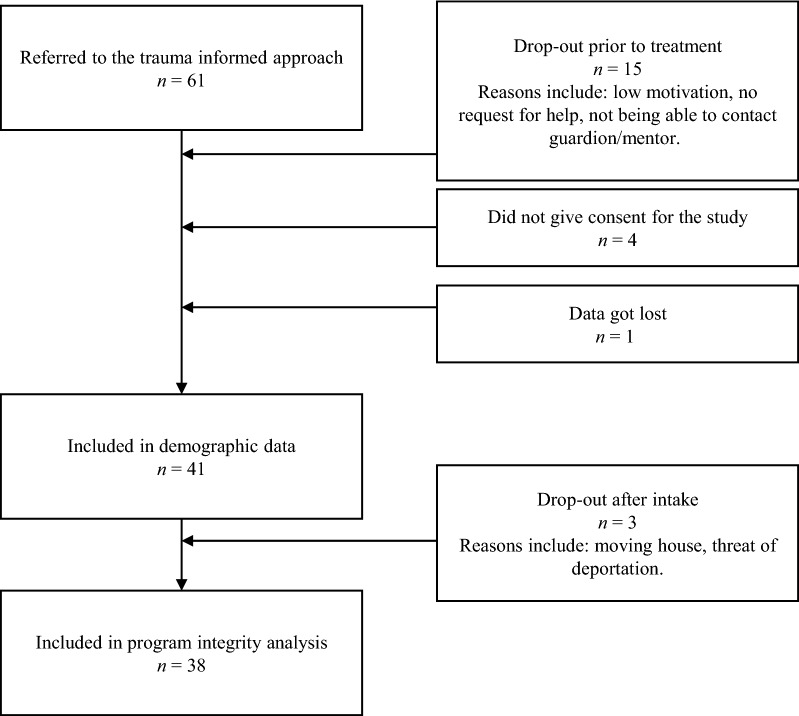
Flowchart of participants

In total, 61 minors were referred to the treatment approach. Fifteen did not start with the approach for a variety of reasons, including: not being able to contact the guardian and/or minor (*n* = 3), not being motivated for treatment (*n* = 3), no longer having a request for help because family reunification took place (*n* = 2), and starting treatment at another facility whilst placed on the waitlist (*n* = 2). In addition, four minors did not give consent to taking part in the study. Ultimately, 41 minors participated in the study. Three participants dropped out immediately after intake, for the following reasons: moving house, needing another form of support because of current threat of deportation, and placement in a secure psychiatric facility. A flowchart is provided in Fig. 2. Demographic characteristics of the URM participants are listed in Table 1. Moreover, all therapists (*n* = 9) and all but one of the ICMs (*n* = 8) completed the online questionnaires. The ICM who did not complete the questionnaire was contacted several times but did not reply to the requests of the researchers.

**Table 1:** Demographics of study participants (N = 41)

Variable*	*n* (%)	M (SD)	Range
Gender (*n* = 41)			
Female	14 (34.1)	–	–
Male	27 (65.9)	–	–
Age (years) (*n* = 41)		16.5 (1.5)	12–19
Country of origin (*n* = 41)			
Eritrea	31 (75.6)	–	–
Syria	9 (22.0)	–	–
Afghanistan	1 (2.4)	–	–
Grew up in town or city (*n* = 15)			
Town	11 (73.3)	–	–
City	4 (26.7)	–	–
Family members in the Netherlands? (*n* = 19)			
Yes	7 (36.8)	–	–
No	12 (63.2)	–	–

*Number of participants for whom data were available are in parentheses

### Request for help

Most participants (95.1%) reported psychological problems, such as difficulties sleeping and concentrating, flashbacks, and feelings of depression. Moreover, psychosocial problems, such as worries concerning family members and problems with housing, were reported by 27 participants (65.9%). Finally, six participants (14.6%) stated that they experienced somatic complaints, such as headaches and incontinence.

### Programme integrity

The data of minors who attended at least one session after intake were included in analyses based on the programme integrity list (*n* = 38). Participants were offered 8 sessions on average (range 4–17), which lasted approximately 80 min. Three minors dropped out after 2 or 3 sessions for the following reasons: wanting to focus on their future and preparing for their asylum interview, not being able to find motivation for treatment, and experiencing too much stress after receiving negative news concerning their asylum status.

Therapists collaborated with ICMs during 93% of the sessions. The main reasons for collaboration with ICMs reported by the therapists included ICMs bridging the cultural and/or language gap (53.7%), that collaboration with ICMs is part of the protocol (16.6%), and ICMs helping in creating a connection with the minors (13.2%). Therapists sometimes worked with professional interpreters (3.3%), or without an ICM or interpreter (3.7%), mostly when the ICM was not able to join a session due to practical reasons. During the majority of the sessions, therapists rated their collaboration with ICMs positively (96.4%), stating for example that they ‘understand the culture’, and that they promote the building of a trusting relationship. In a few cases therapists said the collaboration could be improved—this was mostly when the ICM joined the session online or via telephone as a result of restrictions due to COVID-19.

The sessions focused primarily on continuous stressors (27.1%), trauma (21.9%), laying the lifeline (20.8%), getting to know each other (11.5%), saying goodbye (8.7%), and psychoeducation (7.3%). During 56% of the sessions, therapists followed a treatment module protocol. In these sessions, therapists primarily offered EMDR (47.2% of the sessions) and the lifeline derived from KIDNET (40.3% of the sessions). In other sessions where a protocol of a treatment module was followed, therapists used CBT (11.9% of the sessions following a protocol), or specific protocols, for example focused on stabilization or dealing with anger.

Therapists documented several deviations from the treatment approach. For example, they deviated to address the current needs of the minor, which often concerned dealing with continuous stressors (e.g. worries about family members, problems concerning school and housing), and news the minors had received (e.g. concerning asylum status, family reunification, death of a loved one). Moreover, therapists noted that they sometimes needed to take extra time for psychoeducation, explaining EMDR, building trust, laying down the lifeline and translation. The circumstances due to COVID-19 resulted in several deviations from the approach for approximately a third of the minors. For example, two of the minors received all sessions online/over the telephone. In addition, several minors were contacted online or via telephone by their therapist between sessions for continuity. These sessions resulted in several challenges, including arranging devices, a secure internet connection, and adequate space for the minors. Furthermore, the focus of these sessions sometimes shifted to dealing with social isolation and restrictions. Therapists reported they took time to involve the direct context of the minors, for example by having a guardian or mentor attend the intake interview, and by talking to a guardian or mentor concerning the minors’ continuous stressors. In addition, therapists sometimes deviated from the EMDR protocol because minors reported they did not want to continue, for example because their arousal was too high or because they had an extreme headache.

Therapists described many differences between the participating minors. For example, whereas some minors were very motivated and wanted to talk about their experiences, others had difficulties developing a trusting relationship. One minor stated he did not want to continue with EMDR as it reminded him of magic. In some cases, treatment was mostly focused on motivating the minor to receive mental health care. Treatment termination took place for different reasons. Most minors felt the treatment had helped them overcome their problems (75.1%), some minors were referred to another mental health facility (11.4%), and others wanted to focus on their future/current worries rather than therapy (8.6%).

### Evaluation by therapists

The therapists’ experience with offering the treatment approach varied widely. For example, three therapists had treated one minor, and two others had seen approximately 20 minors. All therapists found the approach useful and wanted to continue offering it. According to the therapists the most important elements of the approach included outreach work, working on a mutual established goal, and working with an ICM with a similar cultural background to the minor. They also valued the flexibility of the approach and being able to deviate to suit the needs of the minor. Some therapists experienced difficulties whilst explaining EMDR to the minors, and some thought that EMDR did not always suit the minors’ needs. Several stated that the protocol could be improved by some adaptations, for example by allowing for more flexibility, and by adding more psychoeducation, CBT exercises relaxation exercises, and homework exercises to the protocol. Finally, they underlined the importance of local professionals throughout the Netherlands offering the approach, and of sharing knowledge and expertise.

*Therapist: ‘We reach minors that wouldn’t normally be reached. We try to adapt to fit to the needs of the minor, instead of the minor adapting to the mental health care institution.’*

#### Evaluation by ICMs

Eight ICMs took part in the evaluation. Their experience with the approach varied from one treatment to more than ten treatments. The majority of ICMs were involved in more than four treatments. Seven ICMs found the approach useful and all ICMs wanted to continue to offer the approach. The ICM who did not find the approach useful stated that more time should be invested in getting to know each other, and that the therapists as well as the ICM should share more personal stories with the minors. ICMs especially valued that the treatment approach could be adapted to fit the minors’ needs better. ICMs rated the KIDNET lifeline, the outreach work, and the engaged approach by the therapist and ICMs as the most important elements of the approach. Most ICMs felt their work was an important addition to the approach. For example, one ICM explained that ICMs can assist in formulating certain questions. Some ICMs added that the approach could be improved by offering more psychoeducation, and spending time on building trust. Several ICMs stated their work could be improved by receiving more training focused on their role in the treatment approach, and some ICMs stated that the communication between therapist and ICM could be improved. Finally, the ICMs highlighted the importance of involving the patient’s context and added that this could be improved.

*ICM: ‘Before the therapy begins, the ICM and therapist should spend more time with the minor. Otherwise, you can’t build trust. My experience is that when more time is invested in the beginning, the outcomes are better’*

#### Course of symptoms

Most participants did not complete the questionnaires. Twelve participants completed the Pre- and post-measurements of the PHQ-A, and 17 completed the pre- and post-measurements of the CRIES-13. Obstacles in obtaining complete measurements included drop-out; limited time to conduct the assessments; restrictions due to COVID-19 (in some cases, therapists and ICMs were not able to explain the questionnaires online or over the telephone); a lack of motivation to fill in the questionnaires; and difficulties with understanding the questions. Another reason for non-completion was that therapists were afraid to burden the minors by asking questions about their mental health.

Based on an independent t-test and a Chi-square test, we found no differences in demographic information (age, gender, country of origin, whether they came from a town or city) or baseline scores on the PHQ-A and CRIES-13 between minors who completed the questionnaires and minors who did not complete the questionnaires. Most participants who filled in the CRIES-13 pre-treatment (24 out of the 29, 82.8%) had developed heightened symptoms of PTSD, and approximately half (12 out of 21, 57.1%) reported moderate to severe symptoms of depression. A paired t-test was conducted to compare pre-and post-intervention scores on the CRIES-13 and PHQ-A (see Table 2). A statistically significant reduction in PTSD scores was found. There was no significant change in depression scores. To ensure robustness, Wilcoxon signed-ranks tests were also conducted, yielding a significant difference for the CRIES-13, but not for the PHQ-A (see Table 2).

**Table 2:** Results of Paired t-test and Wilcoxon Signed-Rank Test Examining the Change in Symptoms

Questionnaire	*n*	M	SD	df	*t*	*p*	Cohen’s *d*	Z	*p*
PHQ-A pre	12	10.35	7.17	10	0.68	0.51	0.20	− 0.59	0.56
PHQ-A post	12	8.82	8.49						
CRIES-13 pre	17	42.59	12.13	16	5.43	< 0.001^*^	1.32	− 3.62	< 0.001^*^
CRIES-13 post	17	19.93	19.13						

*CRIES-13* Children’s Revised Impact of Events Scale, *PHQ-A* Patient Health Questionnaire for Adolescents, *RCI* Reliable Change Index

*p < 0.01

Table 3 shows the CRIES-13 and PHQ-A scores at t_1_ and t_3_, and the RCI scores for participants who completed pre- and post-treatment measurements. As indicated by the RCI, 10 of the 17 participants who completed the CRIES-13 improved (59%), seven remained unchanged (41%), and none worsened. Nine of the participants who improved scored below the cut-off point after treatment. Concerning the PHQ-A, three of the 12 participants improved (25%), eight remained unchanged (67%), and one worsened (8%). Two participants who improved scored below the cut-off score after treatment, whereas two participants who scored below the cut-off point before treatment, scored above the cut-off point after treatment.

**Table 3:** Changes and RCI of symptoms of PTSD and depression

Participant no	CRIES-13 t_1_	CRIES-13 t_3_	RCI CRIES-13 t_1_-t_3_	PHQ-A t_1_	PHQ-A t_3_	RCI PHQ-A t_1_-t_3_
1	49	36	− 1.82	6	18	3.19*
2	30.3	23	− 1.03	27	18	− 2.39*
3	50	32	− 2.52*	14	12.4	− 0.43
4	51	6	− 6.31*	–	–	–
5	13	6	− 0.98	1	3	0.53
6	52	4	− 6.73*	15	1	− 3.72*
7	34	28	− 0.84	5	3	− 0.53
8	53	50	− 0.42	–	–	–
9	49	28.2	− 2.92*	8	12	1.06
10	49	13	− 5.05*	–	–	–
11	27	6	− 2.95*	–	–	–
12	43	0	− 6.03*	–	–	–
13	40.1	0	− 5.62*	12.4	0	− 3.29*
14	46	41	− 0.70	11.3	16	1.26
15	29	5	− 3.37*	3	0	− 0.80
16	61	60.7	− 0.05	16	22.5	1.73
17	47.7	0	− 6.69*	5.63	0	− 1.50

*CRIES-13* Children’s Revised Impact of Events Scale, *PHQ-A*  Patient Health Questionnaire for Adolescents, *RCI*  Reliable Change Index

*Reliable change as indicated by the RCI

## Discussion

The aim of the current study was to provide an evaluation of a multimodal trauma-focused treatment approach offered to URMs in the Netherlands. Forty-one URMs, mostly from Eritrea and Syria, started with the intervention. These minors arrived in the Netherlands after a harrowing journey, often being exposed to atrocities, hunger, thirst, and losing loved ones including companions and friends. Minors reported they would like to receive help to alleviate psychological issues, such as difficulties sleeping. Moreover, most minors reported psychosocial problems, including worries about their family members and their asylum status. A few minors stated they wanted help with somatic issues. Somatization in refugees is connected with psychopathology and can be perceived as an idiom of distress [38]. Moreover, some minors experienced physical abuse and neglect and might have experienced somatic issues as a result. In addition, most minors had developed heightened symptoms of PTSD, and approximately half reported moderate to severe symptoms of depression.

Minors attended approximately eight sessions of the trauma-focused treatment approach. Concerning programme integrity, therapists often deviated from the approach. ICMs and therapists elaborated that they often needed more time to offer psychoeducation. Moreover, therapists and ICMs emphasized the need to adapt the focus of the session to meet the current need of the minors. In addition to a focus on trauma, continuous stressors often played a prominent role in the treatment approach. The treatment approach often consisted of a combination of modules, including the lifeline (as derived from KIDNET), sessions focused on trauma, and sessions focusing on continuous stressors.

The feasibility of the pre- and post-measurements of PTSD and depression was low. The majority of the minors did not complete the questionnaires, and as a result, these findings are not representative of the entire sample. Most minors who did complete the measurements reported a decrease in symptoms of PTSD. No significant change in symptoms of depression was found. An important reason for non-completion was that therapists were afraid to burden the minors by asking questions about their mental health. It is possible the minors who were experiencing mental health issues were spared by not conducting the questionnaires. This finding emphasizes the need of an (independent) assessor other than the therapist to conduct the questionnaires.

The results suggest that the trauma-focused treatment approach is partly feasible and indicates that certain barriers to mental health care can be overcome by offering this short-term, outreach approach. For example, several minors who experienced barriers to regular mental health care, where motivated for referral to another mental health facility after getting offered the treatment approach. The professionals involved in the execution of the treatment approach evaluated it positively, and all of them said they wanted to continue to offer it. Moreover, the drop-out (15% dropped out after 1–3 sessions) was similar to drop-out in other studies researching trauma-focused treatments [47]. Drop-out was often due to circumstances linked to the minors’ asylum status, and needing more help than this short-term treatment approach can offer.

There were several limitations to the feasibility of the treatment approach, including low treatment adherence and challenges faced during implementation of the protocol. Notably, factors limiting programme integrity and feasibility were frequently related to structural barriers. For example, deviations, reasons for drop-out, and missing data, were often due to factors such as news concerning asylum status, moving house, and restrictions due to COVID-19. When evaluating a treatment approach in the context of trauma and resettlement, structural barriers often arise. Although these barriers are out of reach for the minors and therapists, it is important to take these barriers into account while planning the evaluation and organizing funding. For example, it could be helpful to adopt a flexible approach to deal with the ever-changing context of these young refugees, and to support URMs in developing coping strategies to deal with these continuous, structural stressors.

### Strengths and limitations

The current study is one of the first to evaluate a trauma-focused treatment approach specifically for URMs [12, 13]. Although there are several limitations due to the design of the study, this study offers an overview of a treatment approach that can be offered to this understudied population in a naturalistic setting. Conducting this culturally-sensitive outreach approach, we were able to reach a group subject to barriers to mental health care who often do not receive adequate care. Another strength of the current study is that different perspectives are presented, including the perspectives of the therapists, ICMs and URMs.

A factor that might have contributed to the feasibility of the trauma-focused intervention was the flexibility of the treatment approach. During a previous evaluation [11], professionals noted the importance of offering a flexible approach. This study offered insight into the wide range of requests for help reported by the participating URMs. Treatment sessions were often tailored to meet the needs of the minors, which is reflected in the main themes of the sessions as well as in the deviations reported by the therapists. A multimodal approach was conducted, offering modules of KIDNET, EMDR, and CBT, all of which are promising or recommended treatments for PTSD and depression. Multimodal treatments have been offered successfully to children suffering from psychological problems, including depression, anxiety, and trauma [48, 49]. Although the flexible approach was valued as a strength by both therapists and ICMs, it complicates drawing conclusions on what aspects of the approach contributed to its impact.

Several limitations deserve attention. First, the data collection of the questionnaires in this study relied solely on therapists, as there was insufficient funding for researchers to conduct the measurements. Because many sessions did not contain the planned treatment, it is not possible to draw any inferences about the impact of the presented treatment approach. Second, therapists were recruited based on their training level and experience with minors with different cultural backgrounds. It is plausible that this resulted in the selection of highly motivated therapists, and therapists with less experience and affinity with this population might have evaluated the approach less positively. Third, the CRIES-13 was used in the current study to measure symptoms of PTSD, however, this questionnaire is based on the Diagnostic and Statistical Manual of Mental Disorders-IV (DSM-IV). Future studies should include a more up-to-date questionnaire in line with the current DSM-5 or the International Classification of Diseases 11th Revision (ICD-11), such as the Child and Adolescent Trauma Screen (CATS) [50]. Fourth, URMs form a heterogeneous group with a wide range of cultural backgrounds, experiences, and requests for help. The generalizability of the findings is limited, as the current study only focused on a small sample size with participants from three different countries. As a prior evaluation of this approach focused solely on Eritrean minors, guardians often referred Eritrean minors. The current design did not allow for comparison of the treatment’s impact for different cultural groups.

### Research implications

The current study aimed to explore the feasibility of the multimodal trauma-focused treatment approach. Future efforts should focus on examining the effectiveness of this treatment approach. Demazure et al. [12] state that there are many challenges to conducting double-blinded RCTs of the impact of interventions for URMs. However, the authors suggest that small-N designs could be a viable alternative to evaluate intervention effectiveness. In addition to a quantitative evaluation, future research can adopt a mixed-methods approach by including qualitative data, such as interviews with URMs who have received treatment, in order to hear the voices and experiences of the URMs themselves.

### Clinical implications

The collaboration between psychologists and ICMs emerged as one of the strengths of the treatment approach. The involvement of ICMs went beyond the translation of language. ICMs can aid in providing a culturally sensitive explanation of the treatment rationale, in bridging the cultural gap, in building a trusting relationship, and in motivating minors. Nierkens et al. [51] emphasize that the quality and accessibility of health care can benefit from collaborating with ICMs. In line with the evaluation by ICMs, Qureshi et al. [52] note that the role of ICMs can be improved by offering clarity regarding their role. To contribute to the professionalization of ICMs, it is essential to continue to offer supervision, guidance and training.

The current study offers several clinical implications for the multimodal trauma-focused treatment approach. Modifications include providing specific approaches to address continuous stressors, and offering more attention to psychoeducation, as an important deviation from the approach was that therapists often spent more time on continuous stressors and psychoeducation. Continuous stressors related to resettlement can play a crucial role in the development of mental health problems in refugees. A recent pilot study emphasized that current stressors, including the rejection of the asylum claim, can potentially increase symptoms of PTSD in URMs post-treatment. Bruhn et al. [53] state that identifying and dealing with postmigration stressors might limit the impact of these stressors on treatment. Although addressing these problems can be very complex and at times impossible (e.g. when they are related to local legislation), interventions can focus on dealing with these problems, for example by strengthening the social network, and by improving coping strategies. The protocol could therefore benefit from providing specific (CBT) approaches to deal with continuous stressors. In addition, both therapists and ICMs noted they often needed more time for psychoeducation, which psychoeducation is suggested to have a positive impact on PTSD and psychosocial factors, such as social support [54]. The treatment approach protocol can be further improved by offering more attention to psychoeducation.

One of the major future challenges is implementing the presented treatment approach in a sustainable way. The current design requires many resources, including highly trained and experienced ICMs and therapists. Therapists conducting the treatment approach have to be trained to offer KIDNET, EMDR and CBT. However, it is not always feasible to secure these resources. One solution might be to put effort into training and motivating therapists who are less experienced. Motivation for participation in offering this treatment approach might be the collaboration with ICMs and working together with a network of experienced therapists to find out what works for URMs. To assure quality of the treatment, continued supervision and multidisciplinary consultation needs to be offered. Another option is to invest in alternative models. For example, the World Health Organization developed Problem Management Plus (PM +), a brief intervention targeting common mental health problems, based on CBT and problem solving strategies [55]. A recent study found that PM + is acceptable, feasible, safe, and may be effective in improving mental health and psychosocial functioning in refugees. In addition, PM + can be delivered by non-specialist refugee helpers [56]. Furthermore, two RCTs are currently assessing the effect of a stepped care approach addressing PTSD in URMs [57, 58]. Using a stepped care model lay counsellors or ICMs could be trained to offer psychoeducation and the lifeline as derived from KIDNET.

The presented treatment approach seems to be indicated when URMs and their guardians experience barriers to regular mental health care. The outreach care and collaboration with ICMs aids in overcoming these barriers. However, it remains unclear whether the content of the treatment approach—including the lifeline as derived from KIDNET, EMDR, and CBT—is more suitable than other treatment approaches, such as TF-CBT. Future research efforts should focus on what treatment (module) is indicated for which minor. The aforementioned studies focusing on a stepped care model can further inform us on what works.

### Conclusion

This study offers a first evaluation of the feasibility of a multimodal trauma-focused treatment approach specifically for URMs in the Netherlands, taking into account their specific needs; the context; pre- and postmigration stressors; and language and cultural differences. The results are promising and provide a first indication that this approach mostly overcomes barriers to mental health care, and that the treatment is partly feasible.

## Data Availability

Due to the nature of this research, participants of this study did not give consent for their data to be shared publicly, so supporting data is not available.

## Code availability

The SPSS syntax code is available from the corresponding author, CvE, upon reasonable request.

## Declarations

### Clinical trial registration

The study was registered in the Netherlands Trial Register (NL8585), 10 April 2020.

## References

[1] UNHCR. Refugee children. Guidelines on protection and care. Geneva: United Nations High Commissioner for Refugees; 1994.

[2] Unterhitzenberger J, Wintersohl S, Lang M, König J, Rosner R (2019). Providing manualized individual trauma-focused CBT to unaccompanied refugee minors with uncertain residence status: a pilot study. Child Adolesc Psychiatry Ment Health.

[3] Drožđek B, Kamperman AM, Tol WA, Knipscheer JW, Kleber RJ (2014). Seven-year follow-up study of symptoms in asylum seekers and refugees with PTSD treated with trauma-focused groups. J Clin Psychol.

[4] Laban CJ, Gernaat HB, Komproe IH, Van Der Tweel I, De Jong JT (2005). Postmigration living problems and common psychiatric disorders in Iraqi asylum seekers in the Netherlands. J Nerv Ment Dis.

[5] UNHCR. Assistance to unaccompanied refugee minors. 2004.

[6] Bean T, Derluyn I, Eurelings-Bontekoe E, Broekaert E, Spinhoven P (2007). Comparing psychological distress, traumatic stress reactions, and experiences of unaccompanied refugee minors with experiences of adolescents accompanied by parents. J Nerv Ment Dis.

[7] Derluyn I, Broekaert E (2008). Unaccompanied refugee children and adolescents: the glaring contrast between a legal and a psychological perspective. Int J Law Psychiatry.

[8] Jensen TK, Skar AS, Andersson ES, Birkeland MS (2019). Long-term mental health in unaccompanied refugee minors: pre- and post-flight predictors. Eur Child Adolesc Psychiatry.

[9] Fazel M, Reed RV, Panter-Brick C, Stein A (2012). Mental health of displaced and refugee children resettled in high-income countries: risk and protective factors. Lancet.

[10] Vervliet M, Lammertyn J, Broekaert E, Derluyn I (2014). Longitudinal follow-up of the mental health of unaccompanied refugee minors. Eur Child Adolesc Psychiatry.

[11] Van Es CM, Sleijpen M, Mooren T, te Brake H, Ghebreab W, Boelen PA (2019). Eritrean unaccompanied refugee minors in transition: a focused ethnography of challenges and needs. Resi Trea Child Youth.

[12] Demazure G, Gaultier S, Pinsault N (2018). Dealing with difference: a scoping review of psychotherapeutic interventions with unaccompanied refugee minors. Eur Child Adolesc Psychiatry.

[13] Unterhitzenberger J, Eberle-Sejari R, Rassenhofer M, Sukale T, Rosner R, Goldbeck L (2015). Trauma-focused cognitive behavioral therapy with unaccompanied refugee minors: a case series. BMC Psychiatry.

[14] Pfeiffer E, Sachser C, Rohlmann F, Goldbeck L (2018). Effectiveness of a trauma-focused group intervention for young refugees: a randomized controlled trial. J Child Psychol Psychiatry.

[15] Said G, King D (2020). Implementing narrative exposure therapy for unaccompanied asylum-seeking minors with post-traumatic stress disorder: a pilot feasibility report. Clin Child Psychol Psychiatry.

[16] Bean T, Eurelings-Bontekoe E, Mooijaart A, Spinhoven P (2006). Factors associated with mental health service need and utilization among unaccompanied refugee adolescents. Adm Policy Ment Health.

[17] Ni RM (2013). The causes of mistrust amongst asylum seekers and refugees: insights from research with unaccompanied asylum-seeking minors living in the Republic of Ireland. J Refugee Stud.

[18] van Es CM, Sleijpen M, Mooren T, te Brake H, Ghebreab W, Boelen PA (2019). Eritrean unaccompanied refugee minors in transition: a focused ethnography of challenges and needs. Resid Treat Child Youth.

[19] Majumder P, O'Reilly M, Karim K, Vostanis P (2015). 'This doctor, I not trust him, I'm not safe': the perceptions of mental health and services by unaccompanied refugee adolescents. Int J Soc Psychiatry.

[20] Pharos, editor. Factsheet Alleenstaande Minderjarige Vreemdelingen. Utrecht 2019.

[21] Carroll C, Patterson M, Wood S, Booth A, Rick J, Balain S (2007). A conceptual framework for implementation fidelity. Implement Sci.

[22] Perrin S, Meiser-Stedman R, Smith P (2005). The Children's Revised Impact of Event Scale (CRIES): validity as a screening instrument for PTSD. Behav Cogn Psychother.

[23] Smith P, Perrin S, Dyregrov A, Yule W (2003). Principal components analysis of the impact of event scale with children in war. Pers Individ Diff.

[24] Johnson JG, Harris ES, Spitzer RL, Williams JBW (2002). The Patient Healh Questionnaire for adolescents: validation of an instrument for the assessment of mental disorders among adolescent primary care patients. J Adolesc Health.

[25] Manea L, Gilbody S, McMillan D (2012). Optimal cut-off score for diagnosing depression with the Patient Health Questionnaire (PHQ-9): a meta-analysis. CMAJ.

[26] Richardson LP, McCauley E, Grossman DC, McCarty CA, Richards J, Russo JE (2010). Evaluation of the Patient Health Questionnaire-9 Item for detecting major depression among adolescents. Pediatrics.

[27] Al-Amer R, Maneze D, Ramjan L, Villarosa AR, Darwish R, Salamonson Y (2020). Psychometric testing of the Arabic version of the Patient Health Questionnaire among adolescent refugees living in Jordan. Int J Ment Health Nurs.

[28] Van Es CM, Sleijpen M, Ghebreab W, Mooren T (2019). Cultuursensitief werken met alleenstaande jonge vluchtelingen. Kind Adolesc Prakt.

[29] Neuner F, Catani C, Ruf M, Schauer E, Schauer M, Elbert T (2008). Narrative Exposure Therapy for the treatment of traumatized children and adolescents (KidNET): from neurocognitive theory to field intervention. Child Adolesc Psychiatr Clin N Am.

[30] American Psychological Association. Clinical practice guideline for the treatment of PTSD. American Psychological Association; 2017.

[31] Shapiro F, Maxfield L (2002). Eye movement desensitization and reprocessing (EMDR): information processing in the treatment of trauma. J Clin Psychol.

[32] De Roos C, van der Oord S, Zijlstra B, Lucassen S, Perrin S, Emmelkamp P (2017). Comparison of eye movement desensitization and reprocessing therapy, cognitive behavioral writing therapy, and wait-list in pediatric posttraumatic stress disorder following single-incident trauma: a multicenter randomized clinical trial. J Child Psychol Psychiatry.

[33] Oras R, Ezpeleta SCd, Ahmad A (2004). Treatment of traumatized refugee children with eye movement desensitization and reprocessing in a psychodynamic context. Nordic J Psychiatry.

[34] Diehle J, Opmeer BC, Boer F, Mannarino AP, Lindauer RJ (2015). Trauma-focused cognitive behavioral therapy or eye movement desensitization and reprocessing: what works in children with posttraumatic stress symptoms? A randomized controlled trial. Eur Child Adolesc Psychiatry.

[35] NICE. Post-traumatic stress disorder: the managment of PTSD in adults and children in primary and secondary care 2005. 21834189

[36] NICE. Depression in children and young people: identification and management. National Institute for Health and Care Excellence; 2019. 31577402

[37] Cohen JA, Mannarino AP, Kliethermes M, Murray LA (2012). Trauma-focused CBT for youth with complex trauma. Child Abuse Negl.

[38] Rohlof HG, Knipscheer JW, Kleber RJ (2014). Somatization in refugees: a review. Soc Psychiatry Psychiatr Epidemiol.

[39] Stein DJ, Chiu WT, Hwang I, Kessler RC, Sampson N, Alonso J (2010). Cross-national analysis of the associations between traumatic events and suicidal behavior: findings from the WHO World Mental Health Surveys. PLoS ONE.

[40] Thomas DR (2006). A general inductive approach for analyzing qualitative evaluation data. Am J Eval.

[41] Verlinden E, Opmeer BC, Van Meijel EP, Beer R, De Roos C, Bicanic IA (2015). Enhanced screening for posttraumatic stress disorder and comorbid diagnoses in children and adolescents. Eur J Psychotraumatol.

[42] Kroenke K, Spitzer RL, Williams JB, Löwe B (2010). The patient health questionnaire somatic, anxiety, and depressive symptom scales: a systematic review. Gen Hosp Psychiatry.

[43] Kocalevent R-D, Hinz A, Brähler E (2013). Standardization of the depression screener patient health questionnaire (PHQ-9) in the general population. Gen Hosp Psychiatry.

[44] Jacobson NS, Truax P (1991). Clinical significance: a statistical approach to defining meaningful change in psychotherapy research. J Consult Clin Psychol.

[45] Verlinden E, van Meijel EP, Opmeer BC, Beer R, de Roos C, Bicanic IA (2014). Characteristics of the children's revised impact of event scale in a clinically referred Dutch sample. J Trauma Stress.

[46] Kroenke K, Spitzer RL, Williams JB (2001). The PHQ-9: validity of a brief depression severity measure. J Gen Intern Med.

[47] Imel ZE, Laska K, Jakupcak M, Simpson TL (2013). Meta-analysis of dropout in treatments for posttraumatic stress disorder. J Consult Clin Psychol.

[48] Chiu AW, Langer DA, McLeod BD, Har K, Drahota A, Galla BM (2013). Effectiveness of modular CBT for child anxiety in elementary schools. Sch Psychol Q.

[49] Hagen KA, Olseth AR, Laland H, Rognstad K, Apeland A, Askeland E (2019). Evaluating modular approach to therapy for children with anxiety, depression, trauma and conduct problems (MATCH-ADCT) in Norwegian child and adolescent outpatient clinics: study protocol for a randomized controlled trial. Trials.

[50] Sachser C, Berliner L, Holt T, Jensen TK, Jungbluth N, Risch E (2017). International development and psychometric properties of the child and adolescent trauma screen (CATS). J Affect Disord.

[51] Nierkens V, Krumeich A, de Ridder R, van Dongen M (2002). The future of intercultural mediation in Belgium. Patient Educ Couns.

[52] Qureshi A, Ananyeva O, Collazos F, Bhugra D, Ayonrinde O (2021). Intercultural mediation in mental health care. Oxford Textbook of Migrant Psychiatry.

[53] Bruhn M, Rees S, Mohsin M, Silove D, Carlsson J (2018). The range and impact of postmigration stressors during treatment of trauma-affected refugees. J Nerv Ment Dis.

[54] Im H, Jettner JF, Warsame AH, Isse MM, Khoury D, Ross AI (2018). Trauma-informed psychoeducation for Somali refugee youth in urban Kenya: effects on PTSD and psychosocial outcomes. J Child Adolesc Trauma.

[55] Dawson KS, Bryant RA, Harper M, Tay AK, Rahman A, Schafer A (2015). Problem Management Plus (PM+): a WHO transdiagnostic psychological intervention for common mental health problems. World Psychiatry.

[56] de Graaff AM, Cuijpers P, McDaid D, Park A, Woodward A, Bryant RA (2020). Peer-provided Problem Management Plus (PM+) for adult Syrian refugees: a pilot randomised controlled trial on effectiveness and cost-effectiveness. Epidemiol Psychiatr Sci.

[57] Böge K, Karnouk C, Hahn E, Schneider F, Habel U, Banaschewski T (2020). Mental health in refugees and asylum seekers (MEHIRA): study design and methodology of a prospective multicentre randomized controlled trail investigating the effects of a stepped and collaborative care model. Eur Arch Psychiatry Clin Neurosci.

[58] Rosner R, Sachser C, Hornfeck F, Kilian R, Kindler H, Muche R (2020). Improving mental health care for unaccompanied young refugees through a stepped-care approach versus usual care+: study protocol of a cluster randomized controlled hybrid effectiveness implementation trial. Trials.

